# Role of Unilateral Vocal Cord Palsy in Causing Recurrent Tracheobronchial Foreign Bodies

**DOI:** 10.3389/fped.2019.00282

**Published:** 2019-07-24

**Authors:** Laurence Pincet, Karma Lambercy, Kishore Sandu

**Affiliations:** ENT Head and Neck Surgery Department, Centre Hospitalier Universitaire Vaudois, Lausanne, Switzerland

**Keywords:** unilateral vocal cord paralysis, dynamic airway endoscopy, foreign body aspiration, recurrent aspirations, pediatric endoscopy

## Abstract

**Background:** Foreign body (FB) aspiration in children is a frequent condition managed by ENT pediatric surgeons and pediatric pulmonologists.

**Methods:** We present the case of a 20-months-old child who presented with three recurrent episodes of FB aspiration.

**Results:** At the time of FB removal, an initial dynamic examination of the larynx revealed a unilateral vocal cord palsy (UVCP).

**Conclusion:** For recurrent tracheobronchial FB inhalation, we recommend a systematic dynamic airway endoscopy.

## Introduction

The inhalation of foreign bodies (FB) in children is a frequent event in pediatric emergency clinics that are routinely managed by pulmonologists, ENT, and pediatric surgeons. Obstruction of the airway secondary to FB inhalation is a potentially serious cause of morbidity and mortality in children, with reported deaths accounting for 4–7% (1).

In this report, we describe a case of recurrent FB inhalation in a child with right vocal cord paralysis discovered during a dynamic laryngeal examination.

## Case Report

A healthy 20-month-old boy was brought to the emergency department of our hospital with history of FB inhalation and respiratory distress. In the past 12 months, he already had two similar episodes of FB inhalation (peanut and plastic bead) and was treated at a regional hospital before being referred to us on the third occasion. The hospital notes of the past interventions mentioned the FBs lodged in the right main bronchus. In the past, the child had received treatment for asthma with corticosteroids.

**Figure 1:**
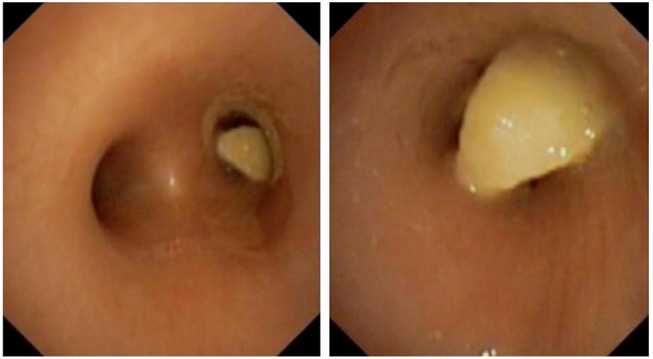
Complete obstruction of the right main stem bronchus by a peanut.

The patient was born at term by a non-complicated delivery, and was in good condition with an age-appropriate development. On examination, the child had dry cough, inspiratory stridor, hoarseness, and hypoventilation of the right lung. The chest X-ray showed an air trapping phenomenon. Due to the past history of recurrent FB inhalation, a dynamic airway endoscopy was performed before the extraction of the FB. Under general anesthesia and the child breathing spontaneously the flexible transnasal laryngoscopy showed a right vocal cord paralysis with the cord lying in an intermediate position (Supplementary Video 1). Subsequent ventilating rigid bronchoscopy showed complete obstruction of the right main stem bronchus by a peanut (Figure 1). The FB was removed using dedicated rigid peanut grasping forceps. The distal airways were normal. In the following days, the child had an uneventful recovery.

In the past history, the child presented with dysphonia and breathy voice since birth. There was no history suggestive of viral infections and surgery. The parents did mention of him occasionally chocking especially with liquids. To complete the investigation for his unilateral vocal cord paralysis, an MRI of the brain, and CT-scan of the neck and chest were performed. They did not show any abnormalities.

Subsequently, the child was given intensive speech and swallow therapy and the parents were educated regarding modifications during feeding. The endoscopy after 1 year showed a complete recovery of the vocal cord paralysis, without any repeat history of FB inhalation.

## Discussion

In this case, our patient presented to the pediatric emergency department with a third episode of FB inhalation. He had history of dysphonia since birth, with occasional chocking episodes but maintained normal growth for his age. This history was taken lightly by the past physicians. This is an error. It is critical to note the exact history of voice change and aspirations in the past of the child, which was missed in our patient by the treating physicians. Our case report highlight the importance of precise history taking, especially of voice change and minor suspicions of aspirations that could be critical in suspecting a vocal cord palsy leading to recurrent FB aspirations.

With recurrent history of FB inhalation, we performed a dynamic examination of the larynx prior to rigid bronchoscopy for extraction of the FB. Dynamic exam showed a right vocal cord paralysis that remained undiagnosed during the two previous endoscopies. On all 3 occasions, the FBs were found lodged in the right main bronchus—possibly meaning that the larger right side incompetent glottis caused the FB to descend straight into the right main bronchus. We report the first case of recurrent FB inhalation secondary to a unilateral vocal cord paralysis (UVCP).

World literature on pediatric airway foreign bodies focuses on the diagnostic approach, missed diagnosis, symptoms of FB inhalation and the value of inspiration-expiration films of chest X-ray. The most frequent symptoms of FB inhalation are cough and chocking, wheezing, and dyspnea (1, 2). But non-specific symptoms (fever, pneumonia, stridor, chest pain, blood stained mucus, restlessness, throat discomfort) or a complete absence of typical symptoms are not unusual (1). Thus, diagnosis of an inhaled FB can be delayed by more than 24 h in about 60% of cases (1). Sometimes undetected FBs might present with complications such as pneumonia, bronchopneumonia, pulmonary edema (1), and sometimes be wrongly treated for bronchial asthma or may even be sent home without treatment following a normal exam and chest X-ray (2). A majority of the FB are found in the right sided bronchus (1).

FB bronchoaspiration while eating should evoke various swallowing disorders that could range from morphological anomalies like laryngotracheal clefts and tracheoesophageal fistulae to neurological disorders in children. These children present early and could have associated comorbidities. In children, 60% of classical FB inhalations (typically peanuts, plastic beads etc.) are reported between 0 and 3 years (1) with an incidence peak between the first and second birthdays. Physiologically, with lack of molar teeth they have less chewing capacity and higher respiratory rates, and hence higher risk to inhale larger food chunks. For classical recurrent FB bronchoaspiration, it is important to consider an eventual underlying pathology.

There are many defensive strategies against bronchoaspiration (3). Safe swallowing implicates precise coordination of cranial nerves and muscles of the oral cavity, pharynx, and proximal esophagus, that are crucial to prevent aspiration into the respiratory passage. During swallowing, the oral phase is a voluntary preparation phase, following which the pharyngeal phase is an involuntary sequence of events. This is the crucial phase in preventing aspirations meaning, aryepiglottic folds cover the glottis, while the false and true vocal folds adduct to complete airway protection. Disorders affecting the central and peripheral nervous system or congenital abnormalities of craniofacial and upper airway structures can affect this protective sphincter and the swallowing mechanism, causing aspirations. Depending on the exact site of aspiration, Wallis (3) described three possible sites and etiologies: (1) Aspirations above the protective sphincter implicates swallowing disorders (e.g., laryngomalacia), (2) those happening through the middle are due to glottic clefts or paralysis, and (3) aspirations occurring below the sphincter are secondary to oeso-gastric causes (e.g., laryngotracheoesophageal clefts and fistulae).

The critical part in the management of recurrent FB inhalation is collaboration with the anesthesia team. In the emergency situation, apart from stabilizing the child's oxygen saturation, it is important to maintain an ideal plane of anesthesia to allow dynamic airway examination. We can then ensure, optimal diagnosis prior to the FB extraction. Although rare, UVCP must kept in mind in such a situation, because of the severe respiratory consequences that will occur if left undetected.

Etiologies of UVCP vary from congenital to acquired causes. Congenital causes include central pathologies, such as Arnold-Chiari malformations, or peripheral pathologies like thoracic vascular or cardiac anomalies (cardio-vocal syndrome). Injury to the peripheral nervous system during prolonged vaginal delivery in new-born infants can cause uni- /bilateral vocal cord palsy (1). The acquired causes include neoplasms of the central nervous system, iatrogenic vagal or recurrent laryngeal nerve trauma secondary to mediastinal surgery (patent ductus arteriosus ligation, repair of tracheoesophageal fistula, or esophageal atresia). Childhood malignancies, such as lymphoma and sarcoma can have a compressive effect on the recurrent or vagus nerve. Infections related to UVCP have also been reported (Epstein-Barr virus, polio, Lyme disease) (1) and must to be ruled out. A dedicated serological and radiological (from the brain up to the thorax) examination helps in correct diagnosis. Needless to say, the treatment is multi- disciplinary and it is critical of all pediatric health care-givers for having the knowledge of such a rare occurrence.

## Conclusion

Our case report highlights the importance of precise history taking especially of voice change and minor aspirations that could be critical in suspecting a vocal cord palsy causing recurrent FB aspiration.

Foreign body inhalation accounts for significant morbidity and mortality amongst infants and small children. In recurrent such episodes, a dynamic airway examination for unilateral vocal cord palsy must be performed.

## Ethics Statement

The study was approved by local institution review board.

## Consent

Written informed consent was obtained from the parents' patient for the publication of the case report and the potentially identifying data and images.

## Author Contributions

LP followed the patient and wrote the case report. KL and KS performed the endoscopy.

### Conflict of Interest Statement

The authors declare that the research was conducted in the absence of any commercial or financial relationships that could be construed as a potential conflict of interest.

## Supplementary Material

Supplementary Video 1 Initial dynamic examination of the larynx reveals a unilateral vocal cord palsy.
